# Shifted Legendre Collocation Method for the Solution of Unsteady Viscous-Ohmic Dissipative Hybrid Ferrofluid Flow over a Cylinder

**DOI:** 10.3390/nano11061512

**Published:** 2021-06-08

**Authors:** Shekar Saranya, Qasem M. Al-Mdallal, Shumaila Javed

**Affiliations:** Department of Mathematical Sciences, United Arab Emirates University, Al-Ain P.O. Box 15551, United Arab Emirates; sannajshekar7@gmail.com (S.S.); shumaila.javed@uaeu.ac.ae (S.J.)

**Keywords:** Legendre collocation method, unsteady, viscous dissipation, Ohmic dissipation, hybrid ferrofluid, cylinder

## Abstract

A numerical treatment for the unsteady viscous-Ohmic dissipative flow of hybrid ferrofluid over a contracting cylinder is provided in this study. The hybrid ferrofluid was prepared by mixing a 50% water (H${}_{2}$O) + 50% ethylene glycol (EG) base fluid with a hybrid combination of magnetite (Fe${}_{3}$O${}_{4})$ and cobalt ferrite (CoFe${}_{2}$O${}_{4})$ ferroparticles. Suitable parameters were considered for the conversion of partial differential equations (PDEs) into ordinary differential equations (ODEs). The numerical solutions were established by expanding the unknowns and employing the truncated series of shifted Legendre polynomials. We begin by collocating the transformed ODEs by setting the collocation points. These collocated equations yield a system of algebraic equations containing shifted Legendre coefficients, which can be obtained by solving this system of equations. The effect of the various influencing parameters on the velocity and temperature flow profiles were plotted graphically and discussed in detail. The effects of the parameters on the skin friction coefficient and heat transfer rates were further presented. From the discussion, we come to the understanding that Eckert number considerably decreases both the skin friction coefficient and the heat transfer rate.

## 1. Introduction

A new nanotechnology-based heat-transfer liquid possessing adequate thermal capabilities is essentially useful for satisfying the needs and demands of manufacturing or innovative firms. Among the prospects are the so-called ferrofluids, which are basically nanofluids that contain a suspension of nanometer-measured solid ferromagnetic particles in customary heat exchange fluids, such as ethylene glycol and water. Ferrofluids exhibit unique fluid attributes and strongly respond to magnetization. Moreover, the mixture of ferroparticles forms a base for various applications in miscellaneous disciplines, for instance, acting as intelligent biomaterials for wound treatment or for use as a medicinal drug aimed at treating cancer and tumors. A range of applications for this type of fluid has been reported by numerous researchers. Dinarvand et al. [1] analyzed the behavior of CuO-Cu/blood hybrid nanofluid flow near the stagnation point of a horizontal porous stretching sheet. It was demonstrated that CuO and Cu hybrid nanoparticles can reduce the capillary’s hemodynamic effect relative to pure blood cases. In addition, the velocity of the blood reduces as the applied magnetic field increases. A new approach for the adaptation of magnetic nanoparticles for the magnetic hyperthermia and the imaging of tumor cells was demonstrated by Vuong et al. [2] using a highly stable ferrofluid based on magnetite nanoparticles, which has high magnetization and high specific absorption. Qi et al. [3] developed a cooling system to reduce CPU temperature using magnetic nanofluids, as increasing heat dissipation is the main factor limiting the function of electronic devices, notably, CPUs. As the intensity of a magnetic field and the rotation angle increase, the results show that the surface temperature of a CPU becomes lower and lower. The thermohydraulic performance of Fe${}_{3}$O${}_{4}$/water-arabic gum (AG) nanofluids in an improved heat exchange system was experimentally explored by Fan et al. [4] to improve the efficiency of heat exchanger systems with a view to reduce the size of equipment and save energy. Mohamed et al. [5] investigated the stagnation point flow and heat transfer properties of Fe${}_{3}$O${}_{4}$/water ferrofluid passed through a stretching sheet with slip effect. Fluid flow past a two-dimensional cylinder carries considerable relevance in many engineering applications. Zaimi et al. [6] applied the Buongiorno’s model to examine the unsteady flow due to a contracting cylinder in a nanofluid. Elnajjar et al. [7] investigated the unsteady fluid flow over a shrinking permeable infinite long cylinder. Results showed that the heat transfer rate increased with the suction parameter. Al-Sakkaf et al. [8] presented an effective iterative power series solution for the unsteady fluid flow over a permeable infinite long cylinder. The validity, accuracy, and efficiency of the current method is verified by comparisons with an exact solution and also with previous methods. Al-Mdallal et al. [9] analyzed the magnetic force effects on the unsteady viscous flow over a shrinking permeable cylinder. Saranya and Al-Mdallal [10] made a comparative study to analyze the performance of three different types of ferroparticles when suspended in a non-Newtonian-type base fluid. The comparative study revealed that a ferrofluid with CoFe${}_{2}$O${}_{4}$ particles has high skin friction rate and a ferrofluid with Ni-ZnFe${}_{2}$O${}_{4}$ particles has high heat transfer rate. Hybrid nanofluid flow over the vertical cylinder by considering shape factor effect was investigated by Hosseinzadeh et al. [11]. Abbas et al. [12] examined the stagnation point flow of hybrid nanofluid over a stretching cylinder. The expanded models for Xue and Yamada Ota were taken into consideration for hybrid nanofluid. Results showed that for control of boundary layer effects in the hybrid nanofluid, an inclined magnetic field is useful.

Several studies have investigated the high thermophysical efficiency of hybrid nanofluids by combining the base fluid with a mixture or composite form of dissimilar suspended nanoparticles. Sundar et al. [13] and Sajid et al. [14] recently published a report detailing the notable composition, thermophysical efficiency, and practical usage of hybrid nanofluids. Devi and Devi [15] studied the dissimilarity, without physical interference, between a hybrid Cu-Al${}_{2}$O${}_{3}$/water nanofluid and a Cu/water nanofluid, revealing that conventional nanofluid heat transport is lower when measured against the hybrid nanofluid. Usman et al. [16] applied nonlinear radiation for an experimental investigation on the same hybrid nanofluid mix, while Maskeen et al. [17] analyzed the improvement of heat transfer in a stretching cylinder with hydromagnetic alumina–copper/water hybrid nanofluid flow. Nadeem and Abbas [18] discovered the magnetohydrodynamics (MHD) and the slip effect in a micropolar hybrid nanofluid passing through a circular cylinder under a stagnation point area. Khashi’ie et al. [19] utilized a permeable circular cylinder as a domain of a thermally stratified flow for a Cu-Al${}_{2}$O${}_{3}$/water hybrid nanofluid. Aminian et al. [20] numerically investigated the effect of a magnetic field on a hybrid nanofluid convection flux in a pore-filled cylinder. Similarly, Kumar et al. [21] explored the effect of an irregular heat source/sink on the thin radiative film flow of an MHD hybrid ferrofluid, whereas Giwa et al. [22] measured the effect of uniform magnetic induction on the heat transport capacity in a rectangular cavity of an aqueous hybrid ferrofluid.

The impact of dissipation in heat-transfer-related issues has been substantially studied. For instance, the enthusiastic applications of viscous dissipation is always worthy of discussion. Temperature rises are often seen in polymer handling streams, i.e., as an infusion trim or an expulsion at top-notch rates. Moreover, streamlined warmth around a swift aircraft in the slight limits increases skin temperature. Numerous warming gadgets—i.e., electric stoves, electric radiators, fastening irons, and cartridge warmers—use Joule warming, whereas it could be utilized by some food handling equipment, provided that Ohmic warming is employed for quick and uniform warming of food items while ensuring the high quality of the ingredients. Theoretical investigations exploring the effects of viscous and Ohmic dissipation on fluids are extensive available in the literature. Suleman et al. [23] addressed the changes in Ag-H${}_{2}$O nanofluid flow over a nonlinear stretching cylinder, incorporating Newtonian heating and homogeneous–heterogeneous reactions. The influence of Joule heating on the radiative nanofluid flow in a semipermeable duct involving the Lorentz forces was analyzed by Li et al. [24]. Kandwal et al. [25] examined the impact of heat generation/absorption and viscous dissipation on the MHD flow of a water-based nanofluid containing silver nanoparticles in an inclined porous cylinder in the presence of suction/injection. Similarly, Mishra and Kumar [26] scrutinized the viscous-Ohmic dissipation effects on nanofluid flow over a stretching cylinder. Khashi’ie et al. [27] investigated the characteristics of a hybrid Cu-Al${}_{2}$O${}_{3}$/water nanofluid due to a radially stretching/shrinking surface with the effects of Joule heating. The similarities and dissimilarities in the flow behavior between hybrid nanofluid and nanofluid with viscous dissipation effect was estimated by Aly and Pop [28]. Chamkha et al. [29] discussed the heat transfer of hybrid nanofluid flow in a rotating system in the presence of thermal radiation and Joule heating.

Inspired by the above literature, the aim of the research is to focus on a theoretical analysis of hybrid ferrofluids by discussing the properties of this mixture under the effect of viscous and Ohmic heating. The hybrid ferrofluid is designed by suspending equal proportion of magnetite (Fe${}_{3}$O${}_{4})$ and cobalt ferrite (CoFe${}_{2}$O${}_{4})$ ferroparticles in an Ethylene glycol (EG)-Water (H${}_{2}$O)-based solution. The base fluid is formed by combining 50% water and 50% ethylene glycol. As far as the authors are aware, there has been no published analysis discussing the influence of viscous-Ohmic dissipation on hybrid ferrofluid flow numerically over an unsteady contracting cylinder using the shifted Legendre collocation method. However, little work has been carried out to study hybrid ferrofluids (see Kumar et al. [21] and Giwa et al. [22]), but still require much attention in order to improve and to realize the full potential of hybrid ferrofluids. From this numerical analysis, we find better opportunity to understand the properties of hybrid ferrofluids under the effect of various parameters. In addition, we initially compare the obtained results with available data in the literature to validate the physical model. The results clearly show excellent accuracy with the results shown in Zaimi et al. [6].

## 2. Problem Formulation

**Figure 1:**
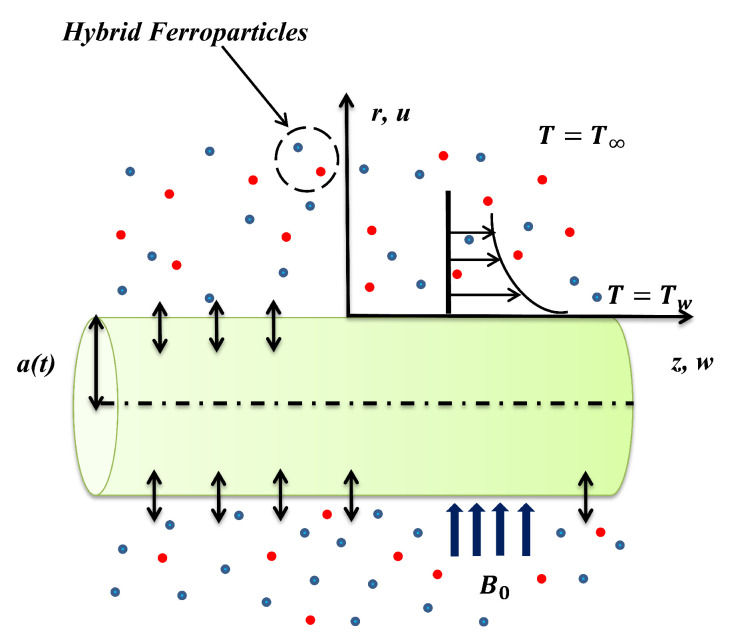
Diagrammatic portrayal of the problem.

Consider an MHD flow of hybrid ferrofluid containing a 50%H${}_{2}$O + 50% EG base fluid with a hybrid combination of magnetite (Fe${}_{3}$O${}_{4})$ and cobalt ferrite (CoFe${}_{2}$O${}_{4})$ ferroparticles. The unsteady contracting cylinder to induce the fluid flow is shown in Figure 1.

A uniform magnetic field of influencing power $B_{0}$ spreads on the fluid flow in the normal direction. Similarly, the unsteady radius of the cylinder $a\left(t\right)=a_{0}\sqrt{1-\gamma t}$ confirms that its diameter is a function of time. Here, the constant γ represents the strength of expansion and contraction, *t* represents the time, and $a_{0}$ represents the positive constant. As the focus of attention is on the viscous and Ohmic dissipation effects, the induced magnetic field effects were neglected in this paper. The cylindrical coordinates *z* were taken along the axial direction of the cylinder, whereas *r* was perpendicular to it.

Table 1 provides a list of the thermophysical properties of nanofluids and hybrid nanofluids. Meanwhile, the applied nanofluid model, the physical meaning of the terms, and the simplifications employed for predicting hybrid nanofluids can be found in Table 2.

The governing flow and heat transfer equations are as follows (Al-Mdallal et al. [9] and Zaimi et al. [6]):

(1) $$\begin{array}{c} \frac{1}{r}\frac{\partial }{\partial r}\left(ru\right)+\frac{\partial w}{\partial z}=0; \end{array}$$

(2) $$\begin{array}{c} \frac{\partial u}{\partial t}+u\frac{\partial u}{\partial r}+w\frac{\partial u}{\partial z}=-\frac{1}{\rho_{nf}}\frac{\partial P}{\partial r}+\nu_{nf}\left(\frac{\partial^{2}u}{\partial r^{2}}+\frac{1}{r}\frac{\partial u}{\partial r}+\frac{\partial^{2}u}{\partial z^{2}}-\frac{u}{r^{2}}\right); \end{array}$$

(3) $$\begin{array}{c} \frac{\partial w}{\partial t}+u\frac{\partial w}{\partial r}+w\frac{\partial w}{\partial z}=-\frac{1}{\rho_{nf}}\frac{\partial P}{\partial z}+\nu_{nf}\left(\frac{\partial^{2}w}{\partial r^{2}}+\frac{1}{r}\frac{\partial w}{\partial r}+\frac{\partial^{2}w}{\partial z^{2}}\right)-\frac{\sigma_{nf}B_{0}^{2}}{\rho_{nf}}w; \end{array}$$

(4) $$\begin{array}{c} \frac{\partial T}{\partial t}+u\frac{\partial T}{\partial r}=\alpha_{nf}\left(\frac{1}{r}\frac{\partial }{\partial r}\left(r\frac{\partial T}{\partial r}\right)\right)+\frac{\mu_{nf}}{{\left(\rho C_{p}\right)}_{nf}}{\left(\frac{\partial w}{\partial r}\right)}^{2}+\frac{\sigma_{nf}}{{\left(\rho C_{p}\right)}_{nf}}B_{0}^{2}w^{2}. \end{array}$$

The boundary conditions for the present model are defined by

(5) $$\begin{array}{c} \left.\begin{array}{c} u=\frac{U}{\sqrt{1-\gamma t}},\;w=-\frac{1}{a_{0}^{2}}\frac{4\nu_{f}z}{1-\gamma t},\;T=T_{w}\;\;\;at\;\;\;r=a\left(t\right),\\ u\to 0,\;\;T\to T_{\infty }\;\;\;as\;\;\;r\to \infty , \end{array}\right\} \end{array}$$

where U(<0), $T_{w}$, and $T_{\infty }$ denote the suction velocity, surface temperature, and ambient temperature, respectively.

At this point, it is necessary to introduce the stream function ψ defined in $u=\frac{1}{r}\frac{\partial \psi }{\partial r}$ and $w=\frac{-1}{r}\frac{\partial \psi }{\partial z}$, as well as the similarity transformations

(6) $$\begin{array}{c} u=-\frac{1}{a_{0}}\frac{2\nu_{f}}{\sqrt{1-\gamma t}}\frac{f(\eta )}{\sqrt{\eta }},\;w=\frac{1}{a_{0}^{2}}\frac{4\nu_{f}z}{1-\gamma t}f^{\prime }\left(\eta \right),\;\;\theta =\frac{T-T_{\infty }}{T_{w}-T_{\infty }},\;\eta ={\left(\frac{r}{a_{0}}\right)}^{2}\frac{1}{1-\gamma t}, \end{array}$$

which automatically follows from the continuity formulation in Equation (1). Thus, Equations (2) and (3) can be reduced to

(7) $$\begin{array}{c} \frac{1}{\left\{\left(1-\phi_{s2}\right)\left[\left(1-\phi_{s1}\right)+\phi_{s1}\left(\frac{\rho_{s1}}{\rho_{f}}\right)\right]+\phi_{s2}\left(\frac{\rho_{s2}}{\rho_{f}}\right)\right\}}\frac{\left(\eta f^{\prime \prime \prime }+f^{\prime \prime }\right)}{{\left(1-\phi_{s1}\right)}^{2.5}{\left(1-\phi_{s2}\right)}^{2.5}}+ff^{\prime \prime }-{f^{\prime }}^{2}\\ -S\left(\eta f^{\prime \prime }+f^{\prime }\right)-\left(\frac{\sigma_{hnf}}{\sigma_{f}}\right)\frac{Mf^{\prime }}{\left(1-\phi_{s2}\right)\left[\left(1-\phi_{s1}\right)+\phi_{s1}\left(\frac{\rho_{s1}}{\rho_{f}}\right)\right]+\phi_{s2}\left(\frac{\rho_{s2}}{\rho_{f}}\right)}=0, \end{array}$$

and

(8) $$\begin{array}{c} \frac{1}{\left(1-\phi_{s2}\right)\left[\left(1-\phi_{s1}\right)+\phi_{s1}\frac{{\left(\rho C_{p}\right)}_{s1}}{{\left(\rho C_{p}\right)}_{f}}\right]+\phi_{s2}\frac{{\left(\rho C_{p}\right)}_{s2}}{{\left(\rho C_{p}\right)}_{f}}}\frac{k_{hnf}}{k_{f}}\left(\eta \theta^{\prime \prime }+\theta^{\prime }\right)-PrS\eta \theta^{\prime }+Prf\theta^{\prime }\\ +\frac{1}{\left(1-\phi_{s2}\right)\left[\left(1-\phi_{s1}\right)+\phi_{s1}\left(\frac{\rho_{s1}}{\rho_{f}}\right)\right]+\phi_{s2}\left(\frac{\rho_{s2}}{\rho_{f}}\right)}PrEc\;\eta \;{f^{\prime \prime }}^{2}\\ +\frac{\frac{\sigma_{hnf}}{\sigma_{f}}}{\left(1-\phi_{s2}\right)\left[\left(1-\phi_{s1}\right)+\phi_{s1}\left(\frac{\rho_{s1}}{\rho_{f}}\right)\right]+\phi_{s2}\left(\frac{\rho_{s2}}{\rho_{f}}\right)}PrEcM{f^{\prime }}^{2}=0. \end{array}$$

Consequently, the conditions at the boundary are modified into

(9) $$\begin{array}{c} \left.\begin{array}{c} f\left(\eta \right)=\lambda ,\;\;\;f^{\prime }\left(\eta \right)=-1,\;\;\;\theta \left(\eta \right)=1,\;\;\;at\;\;\;\eta =1,\\ f^{\prime }\left(\eta \right)=0,\;\;\;\theta \left(\eta \right)=0\;\;\;as\;\;\;\eta \to \infty , \end{array}\right\} \end{array}$$

along with the equation of dimensionless parameters

$$\begin{array}{c} Pr=\frac{{\left(\rho C_{p}\right)}_{f}\nu_{f}}{k_{f}},\;\;S=\frac{a_{0}^{2}\delta }{4\nu_{f}},\;\;M=\frac{\sigma_{f}B_{0}^{2}\left(t\right)a_{0}^{2}}{\rho_{f}\nu_{f}},\\ Ec=\frac{4{\left(\nu_{f}z\right)}^{2}}{a_{0}^{2}{\left(C_{p}\right)}_{f}\left(1-\gamma t\right)\left(T_{w}-T_{\infty }\right)},\;\;\;\lambda =-\frac{a_{0}U}{2\nu_{f}}, \end{array}$$

where Pr is the Prandtl number; and *S*, *M*, and λ are the unsteadiness parameter, the magnetic parameter, and the mass flux parameter, respectively. Herein, assume the values of λ>0 for suction and λ<0 for injection.

For the pressure term, manipulating Equation (2) gives an appropriate expression

(10) $$\begin{array}{c} \frac{P}{\rho_{hnf}}=\mathrm{const}+\nu_{hnf}\left(\frac{\partial u}{\partial r}+\frac{u}{r}\right)-\frac{1}{2}u^{2}+\int \frac{\partial u}{\partial t}dr. \end{array}$$

Moreover, the local skin friction coefficient $\left(C_{fx}\right)$ and the local Nusselt number ($Nu_{x}$) are given by

(11) $$\begin{array}{c} C_{fx}=\frac{\tau_{w}}{\frac{\rho_{hnf}w^{2}}{2}},\;\;Nu_{x}=\frac{a_{0}\sqrt{1-\gamma t}q_{w}}{2k_{f}\left(T_{w}-T_{\infty }\right)}. \end{array}$$

Furthermore, the shear stress $\tau_{w}$ and the wall heat flux $q_{w}$ are given by

(12) $$\begin{array}{c} \tau_{w}=\mu_{hnf}{\left(\frac{\partial w}{\partial r}\right)}_{r=a(t)},\;\;q_{w}=-k_{hnf}{\left(\frac{\partial T}{\partial r}\right)}_{r=a(t)}, \end{array}$$

whereas the dimensionless $C_{fx}$ and $Nu_{x}$ are mathematically expressed as

(13) $$\begin{array}{c} C_{fx}Re_{x}^{1/2}=\frac{f^{\prime \prime }\left(1\right)}{{\left(1-\phi_{s1}\right)}^{2.5}{\left(1-\phi_{s2}\right)}^{2.5}}, \end{array}$$

(14) $$\begin{array}{c} Nu_{x}Re_{x}^{-1/2}=-\left(\frac{k_{hnf}}{k_{f}}\right)\theta^{\prime }\left(1\right). \end{array}$$

## 3. Proposed Method

In this section, we will discuss the numerical method used to solve Equations (7) and (8) subject to the boundary conditions (9). It is well-known that closed forms of the Legendre polynomials $L_{n}\left(t\right)$ of degree *n* on [−1,1] are represented by

(15) Ln(t)=∑k=0nnkn+kkt−12k.

Notice that the set of shifted Legendre polynomials $\left\{L_{0},L_{1},\ldots \right\}$ are orthogonal on [−1,1] with respect to the weight function w(t)=1, i.e.,

(16) $$\begin{array}{c} \int_{0}^{1}L_{n}\left(t\right)L_{m}\left(t\right)dt=\frac{2\delta_{n,m}}{2n+1},\;\;\;n,m\in N, \end{array}$$

where

$$\delta_{n,m}=\left\{\begin{array}{cc} 0, & \mathrm{if}\;\;n\neq m,\\ 1, & \mathrm{if}\;\;n=m. \end{array}\right.$$

Since the domain of Equations (7) and (8) is $\left[1,\eta_{\infty }\right)$, we should use the shifted forms of the Legendre polynomials on $\left[1,\eta_{\infty }\right)$. Therefore, setting $\eta =\frac{\eta_{\infty }+1}{2}+\frac{\eta_{\infty }-1}{2}t$ gives

(17) $$\begin{array}{c} L_{n}^{*}\left(\eta \right)=L_{n}\left(\frac{2\eta -1-\eta_{\infty }}{\eta_{\infty }-1}\right),\;\;\;\eta \in \left[1,\eta_{\infty }\right). \end{array}$$

For the sake of convenience, we may rewrite Equations (7) and (8) in the following forms:

(18) $$\begin{array}{c} f^{\prime \prime \prime }+\frac{1}{\eta }\left\{f^{\prime \prime }+C_{1}C_{2}\left[S\left(\eta f^{\prime \prime }+f^{\prime }\right)+{f^{\prime }}^{2}-ff^{\prime \prime }\right]-C_{3}C_{4}Mf^{\prime }\right\}=0, \end{array}$$

(19) $$\begin{array}{c} \theta^{\prime \prime }+\frac{1}{\eta }\left\{-\theta +C_{4}C_{5}\left[PrS\eta \theta^{\prime }-Prf\theta^{\prime }\right]+\frac{C_{4}}{C_{5}}\left[C_{5}PrEc\eta {\left(f^{\prime \prime }\right)}^{2}+C_{5}C_{3}PrEcM{\left(f^{\prime }\right)}^{2}\right]\right\}=0, \end{array}$$

subject to

(20) $$\begin{array}{c} f\left(1\right)=h_{1}\;\;f^{\prime }\left(1\right)=h_{2},\;\;f^{\prime }\left(\eta_{\infty }\right)=h_{3}\;\;\theta \left(1\right)=h_{4},\;\;\theta \left(\eta_{\infty }\right)=h_{5}. \end{array}$$

Here,

$$\begin{array}{ccc} C_{1} & = & \left\{{\left(1-\phi_{s1}\right)}^{2.5}{\left(1-\phi_{s2}\right)}^{2.5}\right\},\\ C_{2} & = & \left\{\left(1-\phi_{s2}\right)\left[\left(1-\phi_{s1}\right)+\phi_{s1}\left(\frac{\rho_{s1}}{\rho_{f}}\right)\right]+\phi_{s2}\left(\frac{\rho_{s2}}{\rho_{f}}\right)\right\},\\ C_{3} & = & \left(\frac{\sigma_{hnf}}{\sigma_{f}}\right),\\ C_{4} & = & \left\{\left(1-\phi_{s2}\right)\left[\left(1-\phi_{s1}\right)+\phi_{s1}\frac{{\left(\rho C_{p}\right)}_{s1}}{{\left(\rho C_{p}\right)}_{f}}\right]+\phi_{s2}\frac{{\left(\rho C_{p}\right)}_{s2}}{{\left(\rho C_{p}\right)}_{f}}\right\},\\ C_{5} & = & \left(\frac{k_{f}}{k_{hnf}}\right). \end{array}$$

We now express the functions f(η) and θ(η) in terms of shifted Legendre polynomials as follows:

(21) $$\begin{array}{c} f\left(\eta \right)\approx P_{f}\left(\eta \right)=\sum_{i=0}^{N+3}p_{\kappa }L_{\kappa }^{*}\left(\eta \right), \end{array}$$

(22) $$\begin{array}{c} \theta \left(\eta \right)\approx P_{\theta }\left(\eta \right)=\sum_{i=0}^{N+3}q_{\kappa }L_{\kappa }^{*}\left(\eta \right), \end{array}$$

where ${\left\{p_{\kappa }\right\}}_{\kappa =0}^{N+3}$ and ${\left\{q_{\kappa }\right\}}_{\kappa =0}^{N+3}$ are undetermined Legendre coefficients that will be determined later. The associated residuals to (18) and (19) are, respectively, given by

(23) $$\begin{array}{c} R_{f}\left(\eta \right)=P_{f}^{\prime \prime \prime }+\frac{1}{\eta }\left\{P_{f}^{\prime \prime }+C_{1}C_{2}\left[S\left(\eta P_{f}^{\prime \prime }+P_{f}^{\prime }\right)+{\left(P_{f}^{\prime }\right)}^{2}-P_{f}P_{f}^{\prime \prime }\right]-C_{3}C_{4}MP_{f}^{\prime }\right\}, \end{array}$$

(24) $$\begin{array}{ccc} R_{\theta }\left(\eta \right) & = & P_{\theta }^{\prime \prime }+\frac{1}{\eta }\left\{-P_{\theta }+C_{4}C_{5}\left[PrS\eta P_{\theta }^{\prime }-PrP_{f}P_{\theta }^{\prime }\right]\right.\\ & & \left.+\frac{C_{4}}{C_{5}}\left[C_{5}\eta PrEc{\left(P_{f}^{\prime \prime }\right)}^{2}+C_{5}C_{3}PrEcM{\left(P_{f}^{\prime }\right)}^{2}\right]\right\}. \end{array}$$

The unknown coefficients ${\left\{p_{\kappa }\right\}}_{\kappa =0}^{N+3}$ are determined by making the residual $R_{f}\left(\eta \right)$ in (23) vanish at the collocation points $\eta_{j}=1+jh;\;j=1,2,\ldots ,N+1$, where $h=\frac{\eta_{\infty }-1}{N+3}$ represents the uniform step size. In addition, the boundary conditions (20) associated to the function *f* are imposed to have the following equations:

(25) $$\begin{array}{ccc} f\left(1\right)=h_{1}, & \Rightarrow & \sum_{i=0}^{N+3}p_{\kappa }L_{\kappa }^{*}\left(1\right)-h_{1}:=0,\\ f^{\prime }\left(1\right)=h_{2}, & \Rightarrow & \sum_{i=0}^{N+3}p_{\kappa }{\left(L_{\kappa }^{*}\left(1\right)\right)}^{\prime }-h_{2}:=0,\\ f^{\prime }\left(\eta_{\infty }\right)=h_{3}, & \Rightarrow & \sum_{i=0}^{N+3}p_{\kappa }{\left(L_{\kappa }^{*}\left(\eta_{\infty }\right)\right)}^{\prime }-h_{3}:=0. \end{array}$$

On the other hand, the coefficients ${\left\{q_{\kappa }\right\}}_{\kappa =0}^{N+3}$ are determined by making the residual $R_{\theta }\left(\eta \right)$ in (24) vanish at the collocation points, $\eta_{j}$ for j=1,2,…,N+2, and by using the boundary conditions (20) associated to the function θ, obtaining

(26) $$\begin{array}{ccc} \theta \left(1\right)=h_{4} & \Rightarrow & \sum_{i=0}^{N+3}q_{\kappa }L_{\kappa }^{*}\left(1\right)-h_{4}:=0, \end{array}$$

(27) $$\begin{array}{ccc} \theta \left(\eta_{\infty }\right)=h_{5} & \Rightarrow & \sum_{i=0}^{N+3}q_{\kappa }L_{\kappa }^{*}\left(\eta_{\infty }\right)-h_{5}:=0. \end{array}$$

In summary, the determination of the coefficients ${\left\{p_{\kappa }\right\}}_{\kappa =0}^{N+3}$ and ${\left\{q_{\kappa }\right\}}_{\kappa =0}^{N+3}$ requires solving the below system of algebraic equations, which consist of 2N+8 equations with 2N+8 unknowns:

(28) $$H\left(V\right)=\left[\begin{array}{c} F(V)\\ G(V) \end{array}\right]:=0,$$

where $V={\left[p_{0},\cdots ,p_{N+3},q_{0},\cdots ,p_{N+3}\right]}^{T}$ consists of all the unknowns. The vectors $F\left(V\right)={\left[F_{0},F_{1},\ldots ,F_{N+31}\right]}^{T}$ and $G\left(V\right)={\left[G_{0},G_{1},\ldots ,G_{N+3}\right]}^{T}$ are, respectively, defined as

$$\begin{array}{ccc} F_{0} & = & P_{f}\left(1\right)-h_{1},\\ F_{j} & = & R_{f}\left(\eta_{j}\right),\;\;\;j=1,\ldots ,N+1,\\ F_{N+2} & = & P_{f}^{\prime }\left(1\right)-h_{2},\\ F_{N+3} & = & P_{f}^{\prime }\left(\eta_{\infty }\right)-h_{3}, \end{array}$$

and

$$\begin{array}{ccc} G_{0} & = & P_{\theta }\left(1\right)-h_{4},\\ G_{j} & = & R_{\theta }\left(\eta_{j}\right),\;\;\;j=1,\ldots ,N+2,\\ G_{N+3} & = & P_{\theta }\left(\eta_{\infty }\right)-h_{5}. \end{array}$$

It should be noted that we use the multidimensional version of Newton’s method to solve (28) by applying the functional iteration procedure, evolved from selecting $V^{0}$ and generating, for s≥1,

(29) $$\begin{array}{c} V^{s}=V^{s-1}-J_{H}{\left(V^{s-1}\right)}^{1}F\left(V^{s-1}\right), \end{array}$$

where $J_{H}\left(V\right)$ represents the Jacobian matrix of H. It is important to mention here that the multidimensional Newton’s method converges quadratically if

(a) $∥J_{H}^{-1}∥\leq M$, where M>0, and the norm of the inverse of the Jacobian at $V^{s}$ is bounded;
(b) $∥J\left(z_{2}\right)-J\left(z_{1}\right)∥\leq ∥z_{2}-z_{1}∥$, the Jacobian is Lipschitz continuous.

## 4. Error Estimates and Convergence Analysis of the Shifted Legendre Collocation Method

In this section, we give some estimates for the error based on the shifted Legendre collocation method and also a bound on the error between the approximate and exact solution.

For that, we apply the method presented in Section 3 to solve Equation (18), which can be written as

(30) $$\begin{array}{c} f^{\prime \prime \prime }+\frac{1}{\eta }\left\{f^{\prime \prime }+C_{1}C_{2}\left[S\left(\eta f^{\prime \prime }+f^{\prime }\right)+{f^{\prime }}^{2}-ff^{\prime \prime }\right]-C_{3}C_{4}Mf^{\prime }\right\}=0, \end{array}$$

with the condition

(31) $$\begin{array}{c} f\left(1\right)=\lambda ,\;\;f^{\prime }\left(1\right)=-1,\;\;f^{\prime }\left(\eta_{\infty }\right)=0. \end{array}$$

To derive the shifted Legendre collocation solution for Equation (30), we first divide the interval $\left[1,\eta_{\infty }\right)$ into a uniform mesh consisting of the collocation points $\eta_{j}=1+jh;\;j=1,2,\ldots ,N+1$, where $h=\frac{\eta_{\infty }-1}{N+3}$ represents the uniform step size.

**Figure 2:**
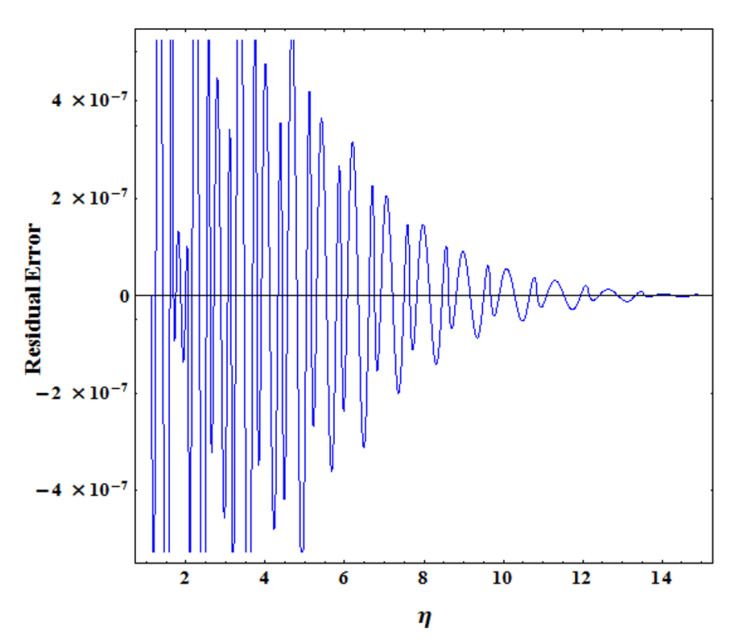
Convergence of residual error for $R_{f}\left(\eta_{j}\right)$ at each collocation point—$\eta_{j}$, j=1,2,…,N+1.

These unknown coefficient ${\left\{p_{\kappa }\right\}}_{\kappa =0}^{N+3}$ are found by expanding them in terms of truncated shifted Legendre polynomials presented in Section 3. A suitable domain truncation value for $\eta_{\infty }$ is determined. Usually, the accuracy of results is insensitive to the near-suitable values of $\eta_{\infty }$, but the results do not change significantly. Figure 2 represents the the convergence of the residual error estimate for $R_{f}\left(\eta_{j}\right)$ at each collocation point—$\eta_{j}$, j=1,2,…,N+1. For fixed values h=0.01 and N=5, the errors decay as η increases.

In Table 3 and Table 4 for the case of suction and injection, we present a comparison between the numerical results obtained for f(η) using the shifted Legendre collocation method with the exact solutions for different values of η and λ. Similarly, in Table 5 and Table 6, for the case of suction and injection, the numerical values of $f^{\prime }\left(\eta \right)$ are compared with the approximate solution obtained using the fourth-order Runge–Kutta method for various values of η and λ.

It is seen from the tables that for fixed h(=0.01) and N(=5), the errors decay as η increases. So, the smoother the exact solutions, the smaller the numerical errors.

## 5. Validation

We initially compare the available data in Zaimi et al. [6] to validate the physical model in (18) and (19). The results clearly show excellent accuracy with the results shown in Zaimi et al. [6]. Table 3 is presented to show the numerical values of skin friction coefficient $f^{\prime \prime }\left(1\right)$. The values are compared for each case of mass flux parameter (λ). Table 7 provides clear proof that when nanoparticle concentration, $\phi_{s1}$ (Fe_3_O_4_), and $\phi_{s2}$ (CoFe_2_O_4_) are not considered, our calculations yield the same results as shown in Zaimi et al. [6].

## 6. Results and Discussion

The graphical data of the parameters active on the common profiles, the local skin friction coefficient, and the rate of heat transfer for the hybrid ferrofluid are discussed in this section. Distinct parameters were calculated by assigning fixed values of S=−1,M=0.5,λ=1 (suction) and λ=−1 (injection), Ec=1, except when a particular parameter is varied to study its effect. For the entire discussion, $f^{\prime }\left(\eta \right)$ and θ(η) are used to mention the velocity and temperature profiles. The figures are discussed for the two mass flux parameter cases (λ>0andλ<0), denoting suction and injection, respectively.

**Figure 3:**
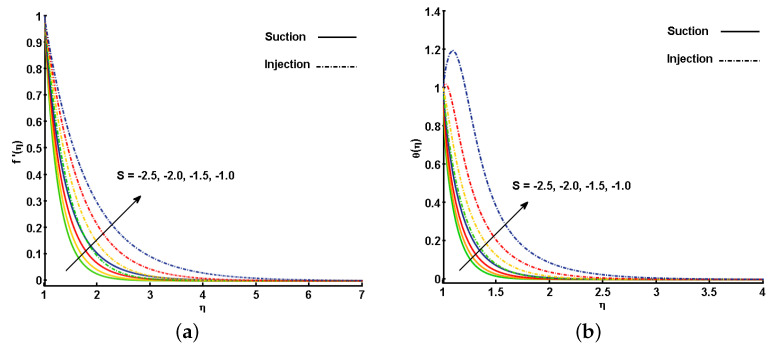
Effect of unsteadiness parameter (S) on (**a**) velocity profile $\left(f^{\prime }\left(\eta \right)\right)$ and (**b**) temperature profile (θ(η)).

Figure 3a,b describe the behavior of the unsteadiness parameter (*S*) on $f^{\prime }\left(\eta \right)$ and θ(η). Note that increasing the values for *S* leads to an increment in the fluid velocity, $f^{\prime }\left(\eta \right)$. This is because when we enhance the values of *S*, there is an enhancement in the momentum boundary layer thickness in response, which eventually helps in the increment of $f^{\prime }\left(\eta \right)$. Similarly, θ(η) is also increased by increasing the values of *S*. The improvement in the thermal boundary layer thickness cause a variation in the temperature distribution all over the domain. This variation reflects positively in the increment of θ(η). It is also worth mentioning that the effect for the mass injection case (λ<0) are more prominent than for the mass suction case (λ>0). The difference between the cases can be clearly seen in Figure 3a,b.

**Figure 4:**
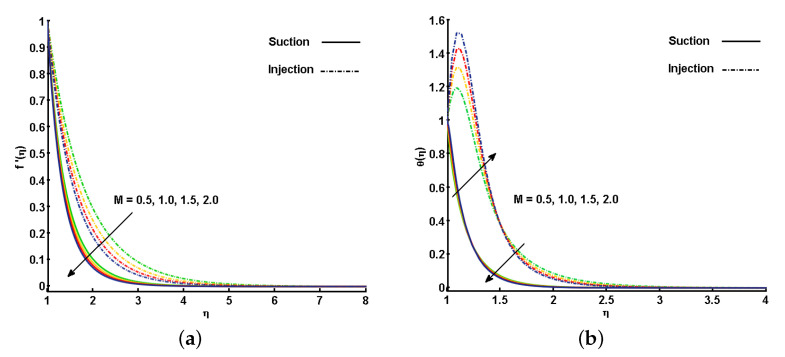
Effect of magnetic parameter (M) on (**a**) velocity profile $\left(f^{\prime }\left(\eta \right)\right)$ and (**b**) temperature profile (θ(η)).

Figure 4a,b illustrate the effect of the different estimations of the magnetic parameter (*M*) on $f^{\prime }\left(\eta \right)$ and θ(η). An increase in *M* corresponds to a decrease in the momentum boundary layer thickness, prompting a decrease in the velocity profile. The reason is that the magnetic field applied in the direction normal to the fluid flow helps in the development of Lorentz force, which is responsible for slowing down the fluid velocity (see Figure 4a). On the other hand, the utilization of the transverse magnetic field in an electrically conducting fluid indicates the highest degree of Lorentz-induced power, which offers sufficient opportunity to the increment in θ(η). As depicted in Figure 4b, θ(η) is increased close the cylinder surface, and as it moves towards the ambient region, an opposite trend is noted. This is because far from the cylinder surface, the velocity is very small, and hence, the induced force of Lorentz is also very small. The effect of the magnetic field on the free stream region is smaller, thus, θ(η) is decreased.

**Figure 5:**
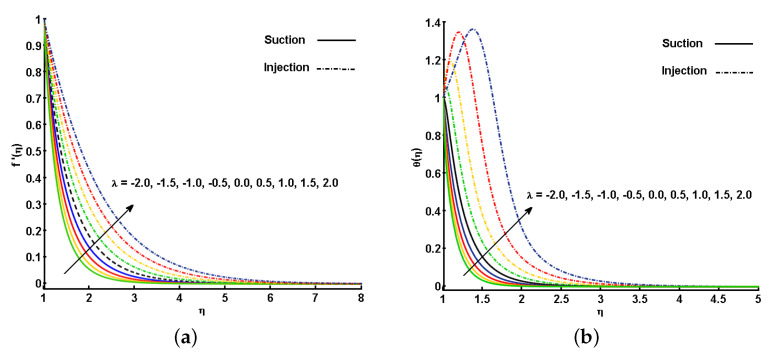
Effect of mass flux parameter (λ) on (**a**) velocity profile $\left(f^{\prime }\left(\eta \right)\right)$ and (**b**) temperature profile (θ(η)).

Figure 5a,b display the behavior of the mass flux parameter (λ>0&λ<0) for the non-dimensional velocity and temperature distributions for both injection and suction case. These curves expose that the $f^{\prime }\left(\eta \right)$ and θ(η) got enhanced for an increment in λ. These figures also show that the injection case results are higher than the suction case. When there is injection/suction in the domain, the heated fluid can be moved further off the wall to accelerate the flow with less viscosity influence. This effect increases the shear by increasing the maximum velocity within the domain (see Figure 5a). In the same way, an increase in λ leads to considerable increase in reactions and viscous source conditions, and consequently the fluid temperature increases significantly (see Figure 5b).

**Figure 6:**
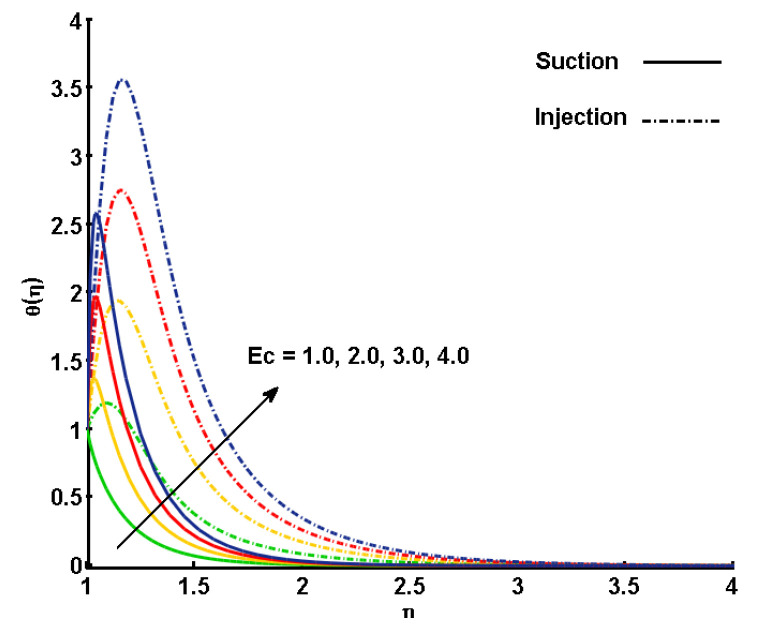
Effect of Eckert number (Ec) on temperature profile (θ(η)).

Figure 6 illustrates the varying effects of the Eckert number (Ec) in the thermal field. As shown, the nondimensional profile of temperature is enhanced with an increment in Ec for the suction and injection cases. Eckert number can play an important role in the process of heat transfer as it measures the kinetic energy relative to the enthalpy difference, which can help in determining the temperature distribution of the flow in the overall domain. For Ec<<1, the effects of viscous dissipation, pressure changes, and body forces can be neglected, since the energy equation reduces to a balance between conduction and convection. With increasing Ec, the effects of dissipation due to internal friction of the fluid are enhanced, by which θ(η) is increased.

**Figure 7:**
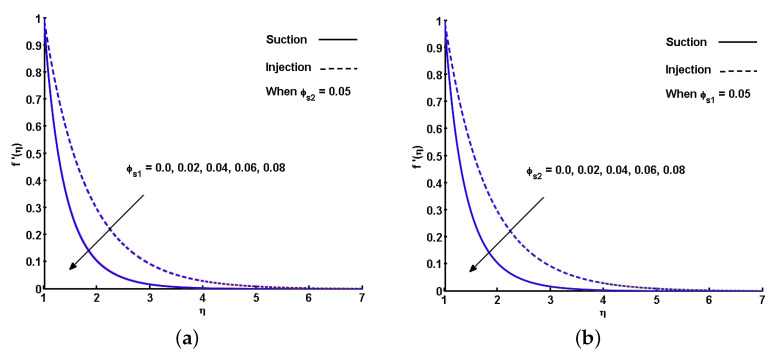
Effect of volume fraction of nanoparticles ($\phi_{s1}$ and $\phi_{s2}$) on velocity profile $\left(f^{\prime }\left(\eta \right)\right)$ (**a**) $\phi_{s1}$ is varied and $\phi_{s2}$ is fixed; (**b**) $\phi_{s1}$ is fixed and $\phi_{s2}$ is varied.

**Figure 8:**
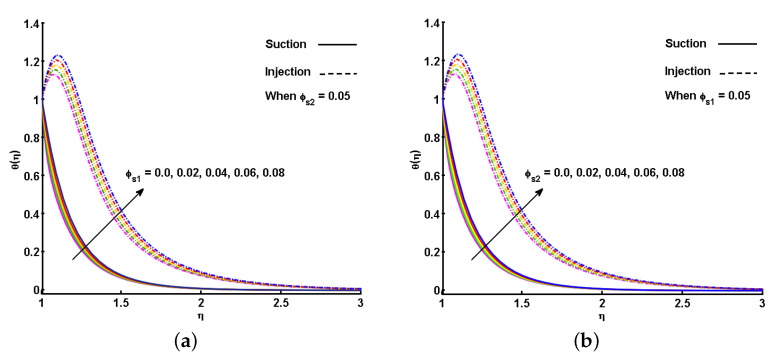
Effect of volume fraction of nanoparticles ($\phi_{s1}$ and $\phi_{s2}$) on temperature profile (θ(η)) (**a**) $\phi_{s1}$ is varied and $\phi_{s2}$ is fixed; (**b**) $\phi_{s1}$ is fixed and $\phi_{s2}$ is varied.

The effect of the $\phi_{s1}$ (Fe${}_{3}$O${}_{4}$) and $\phi_{s2}$ (CoFe${}_{2}$O${}_{4}$) ferroparticles on $f^{\prime }\left(\eta \right)$ is individually portrayed in Figure 7a,b. Increasing $\phi_{s1}$ and $\phi_{s2}$ causes a drop in the velocity profiles of the hybrid ferrofluid for both the suction and injection cases. The impact of $\phi_{s1}$ (Fe${}_{3}$O${}_{4}$) and $\phi_{s2}$ (CoFe${}_{2}$O${}_{4}$) on the dimensionless profiles of temperature is outlined in Figure 8a,b. By gradually increasing the values of $\phi_{s1}$ and $\phi_{s2}$, θ(η) is improved. This gradual increase is due to the augmentation in the thermal boundary layer with increased nanoparticle volume fraction. However, as the concentration surpasses the maximum level, sedimentation occurs. In particular, an impact could not be expected when the volume fraction of Fe${}_{3}$O${}_{4}$ or CoFe${}_{2}$ O${}_{4}$ ferroparticles surpasses 8%. Compared to the suction, the rise in temperature profile is more for injection, with respect to each estimation of $\phi_{s1}$ and $\phi_{s2}$.

**Figure 9:**
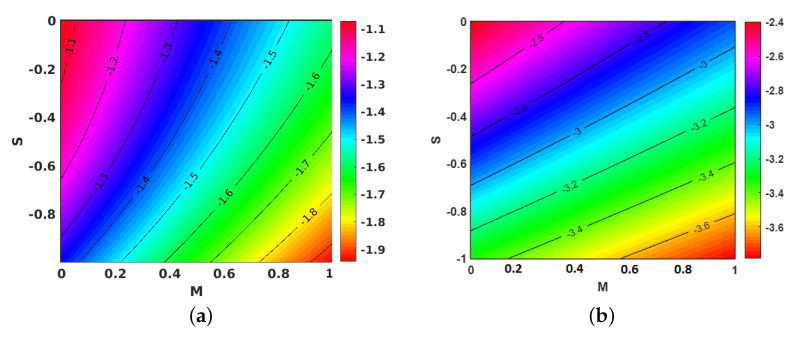
Effect of unsteadiness parameter (S) and magnetic parameter (M) on $f^{\prime \prime }\left(1\right)$. (**a**) Injection; (**b**) suction.

Figure 9a,b portray the impacts of *S* plotted against *M* on the $f^{\prime \prime }\left(1\right)$ for the injection and suction cases, respectively. Gradual increases in *S* relative to *M* caused a decline in the velocity gradient, which can be explained by the increase in the momentum boundary layer thickness for higher values of *S* against *M*.

**Figure 10:**
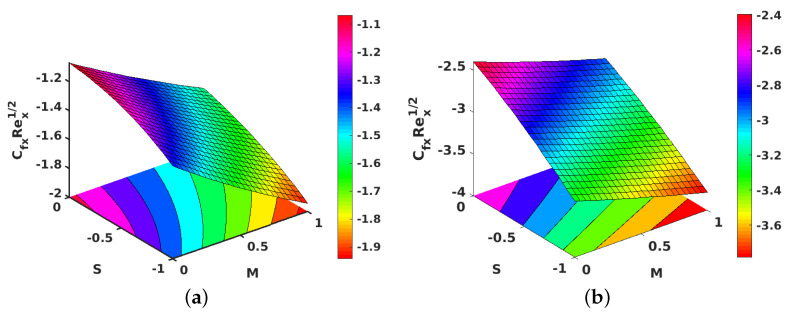
Effect of unsteadiness parameter (S) and magnetic parameter (M) on the local skin friction coefficient. (**a**) Injection; (**b**) suction.

**Figure 11:**
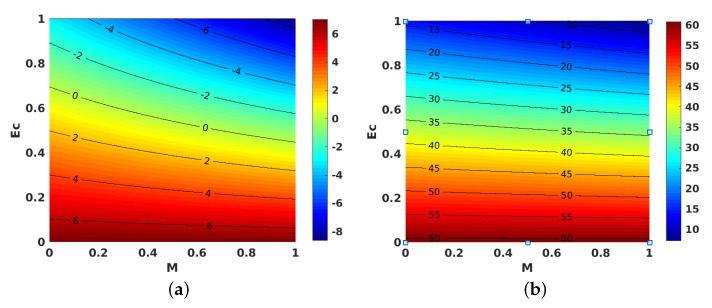
Effect of Eckert number (Ec) and magnetic parameter (M) on $\theta^{\prime }\left(1\right)$. (**a**) Injection; (**b**) suction.

**Figure 12:**
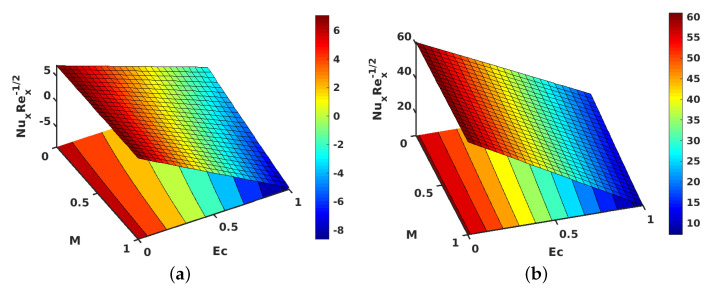
Effect of Eckert number (Ec) and magnetic parameter (M) on local Nusselt number. (**a**) Injection; (**b**) suction.

Figure 10a,b shows the contours for the impact of *S* along with *M*, on the local skin friction coefficient for suction and injection. Figure 11a,b project the variations in $\theta^{\prime }\left(1\right)$ when *M* and Ec are improved. However, improving Ec with *M* yielded a decrement in the values of $\theta^{\prime }\left(1\right)$ for both injection and suction. Similarly, contours for the local Nusselt number with the parameters Ec and *M* are shown in Figure 12a,b for injection and suction.

**Figure 13:**
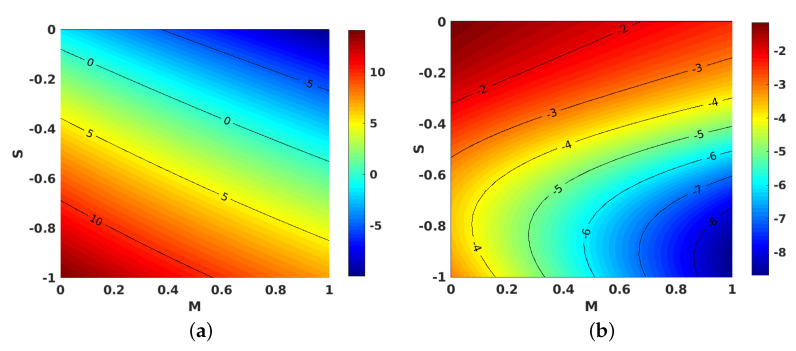
Effect of unsteadiness parameter (S) and magnetic parameter (M) on $\theta^{\prime }\left(1\right)$. (**a**) Injection; (**b**) suction.

**Figure 14:**
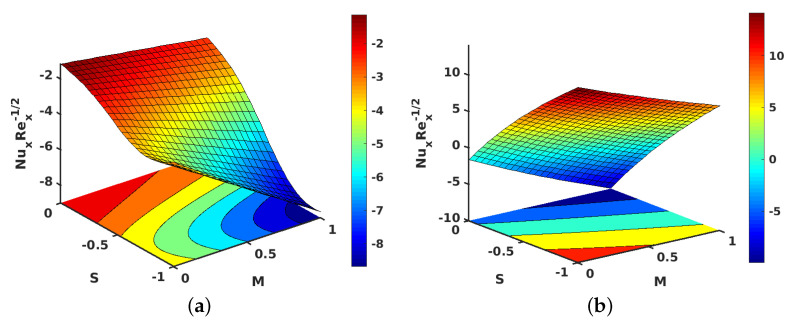
Effect of unsteadiness parameter (S) and magnetic parameter (M) on the local Nusselt number. (**a**) Injection; (**b**) suction.

Figure 13a,b illustrate the behavior of *S* contrasted with *M* on $\theta^{\prime }\left(1\right)$ for both injection and suction. Apparently, $\theta^{\prime }\left(1\right)$ displayed contrasting behaviors in the injection and suction cases. $\theta^{\prime }\left(1\right)$ is decreased when *M* is changed from 0 to 1 and *S* is changed from −1 to 0 for injection. But for suction, $\theta^{\prime }\left(1\right)$ is increased when the changes in parameters *M* and *S* are performed in the same manner. The contours for the same parameters *S* and *M* with the local Nusselt number is provided in Figure 14a,b for injection and suction.

**Figure 15:**
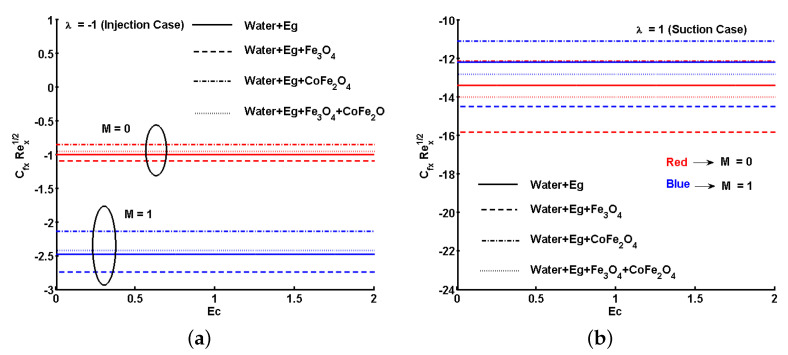
Effect of Eckert number (Ec) and magnetic parameter (M) on the local skin friction coefficient. (**a**) Injection; (**b**) suction.

**Figure 16:**
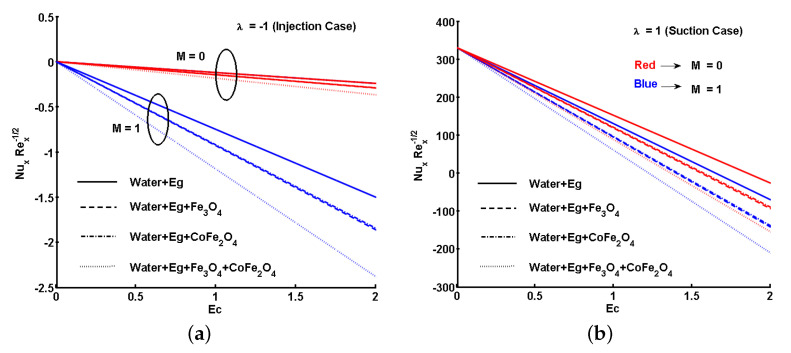
Effect of Eckert number (Ec) and magnetic parameter (M) on the local Nusselt number. (**a**) Injection; (**b**) suction.

Furthermore, Figure 15a,b and Figure 16a,b show the variations for the local skin friction coefficient and the local Nusselt number assuming different fluids cases. The plots were separately inspected and discussed for suction and injection in the presence and absence of *M*. The first case was for a regular fluid, where $\phi_{s1}=0$ and $\phi_{s2}=0$. The second and third cases were for ferrofluids, where $\phi_{s1}=0.05$ (Fe_3_O_4_) and $\phi_{s2}=0$, and $\phi_{s1}=0$ and $\phi_{s2}=0.05$ (CoFe_2_O_4_), respectively. The final case is for hybrid ferrofluid, $\phi_{s1}=0.05$ and $\phi_{s2}=0.05$. Comparing Figure 15a,b, the magnitude of skin friction coefficient is different for each case. That is, the magnitude of skin friction coefficient is less for the case of suction in the absence of *M*. Similarly, we note comparatively different ranges of heat transfer rate from Figure 16a,b, where the rate of heat transfer is high for the suction case in the absence of *M*.

The effects of viscous and Ohmic heating were clearly depicted for each type of fluid. From Figure 15a,b and Figure 16a,b, it could be noted that viscous and Ohmic heating effects do not aid the hybrid ferrofluid to give a better heat transfer rate, mainly because of the concentration of ferroparticles. This is because most of the heat transferred was observed by the ferroparticles and partially transmitted to the base fluid.

Table 8 provides a summary of estimations for $f^{\prime \prime }\left(1\right)$ and $\theta^{\prime }\left(1\right)$ given the various $\phi_{s1}$ and $\phi_{s2}$ conditions in the presence and absence of *M* and Ec. Here, the unsteadiness parameter was fixed at S=−1. Viscous and Ohmic heating considerably reduced $f^{\prime }\left(1\right)$ and $\theta^{\prime }\left(1\right)$ for both suction and injection.

## 7. Conclusions

The study numerically investigates the influence of viscous-Ohmic dissipation on the hybrid ferrofluid flow over an unsteady contracting cylinder using the shifted Legendre collocation method. The effects of the associated parameters on the velocity, temperature field, local skin friction coefficient, and local Nusselt number were examined and illustrated graphically for suction λ>0 and injection λ<0 cases. The main findings of this study can be summarized as follows:

- A decline in the velocity profile is noted with increasing values of *M*, $\phi_{s1}$, and $\phi_{s2}$, whereas the velocity profile is increased for large values of *S* and λ.
- Improvements in *S* and *M* minimized the local skin friction coefficient.
- For increasing *M*, the temperature profile increased in the region close to the cylinder surface and decreased far away from the surface. Whereas, increases in Ec, $\phi_{s1}$, and $\phi_{s2}$ enhanced the temperature profile.
- The magnitude of the local Nusselt number decreased as the values of *S* and *M* intensified.
- Viscous-Ohmic dissipation tends to lower the skin friction coefficient and the heat transfer rate of the hybrid ferrofluid.

Hybrid nanofluid finds applications in different fields such as solar energy, heat pipes, automotive industry, manufacturing industry, heat exchangers, cooling of electronic equipment, etc. Therefore, this topic is more popular among the young scholars and experts working in the field of heat transfer, as more research in this field is required. With the help of present study, we endeavored to identify the challenges in utilizing hybrid ferrofluids by discussing the effects of viscous-Ohmic dissipation.

## Figures and Tables

**Table 1:** Thermophysical properties of the fluid and solid materials in this study (Kumar et al. [21]).

Properties	Water+EG (f)	Fe${}_{3}$O${}_{4}\;\left(s1\right)$	CoFe${}_{2}$O${}_{4}\left(s2\right)$
ρ(kg m${}^{-3}$)	1056	5180	4907
$C_{p}$(J kg${}^{-1}$ K${}^{-1})$	3288	670	700
*k*(Wm${}^{-1}$K${}^{-1})$	0.425	9.7	3.7
σ(Sm${}^{-1}$)	0.00509	0.74 $\times \;10^{6}$	1.1 $\times \;10^{6}$
Pr	29.86	-	-

**Table 2:** Properties of nanofluids and hybrid nanofluids (Devi and Devi [15]).

Properties	Nanofluid	Hybrid Nanofluid
Density	$\rho_{nf}=\left(1-\phi \right)\rho_{f}+\phi \rho_{s}$	$\rho_{hnf}=\left\{\left(1-\phi_{s2}\right)\left[\left(1-\phi_{s1}\right)\rho_{f}+\phi_{s1}\rho_{s1}\right]\right\}+\phi_{s2}\rho_{s2}$
Heat capacitance	${\left(\rho C_{p}\right)}_{nf}=\left(1-\phi \right){\left(\rho C_{p}\right)}_{f}+\phi {\left(\rho C_{p}\right)}_{s}$	${\left(\rho C_{p}\right)}_{hnf}=\left\{\left(1-\phi_{s2}\right)\left[\left(1-\phi_{s1}\right){\left(\rho C_{p}\right)}_{f}+\phi_{s1}{\left(\rho C_{p}\right)}_{s1}\right]\right\}+\phi_{s2}{\left(\rho C_{p}\right)}_{s2}$
Kinematic viscosity	$\nu_{nf}=\frac{\mu_{nf}}{\rho_{nf}}$	$\nu_{hnf}=\frac{\mu_{hnf}}{\rho_{hnf}}$
Dynamic viscosity	$\mu_{nf}=\frac{\mu_{f}}{{\left(1-\phi \right)}^{2.5}}$	$\mu_{hnf}=\frac{\mu_{f}}{{\left(1-\phi_{s1}\right)}^{2.5}{\left(1-\phi_{s2}\right)}^{2.5}}$
Thermal conductivity	$\frac{k_{nf}}{k_{f}}=\frac{k_{s}+\left(n-1\right)k_{f}-\left(n-1\right)\phi \left(k_{f}-k_{s}\right)}{k_{s}+\left(n-1\right)k_{f}+\phi \left(k_{f}-k_{s}\right)}$	$\frac{k_{hnf}}{k_{bf}}=\frac{k_{s2}+\left(n-1\right)k_{bf}-\left(n-1\right)\phi_{s2}\left(k_{bf}-k_{s2}\right)}{k_{s2}+\left(n-1\right)k_{bf}+\phi_{s2}\left(k_{bf}-k_{s2}\right)}$,
		where $\frac{k_{bf}}{k_{f}}=\frac{k_{s1}+\left(n-1\right)k_{f}-\left(n-1\right)\phi_{s1}\left(k_{f}-k_{s1}\right)}{k_{s1}+\left(n-1\right)k_{f}+\phi_{s1}\left(k_{f}-k_{s1}\right)}$
Electrical Conductivity	$\frac{\sigma_{nf}}{\sigma_{f}}=1+\frac{3(\sigma -1)\phi }{(\sigma +2)-(\sigma -1)\phi }$,	$\frac{\sigma_{hnf}}{\sigma_{bf}}=\frac{\sigma_{s2}+2\sigma_{bf}-2\phi_{s2}\left(\sigma_{bf}-\sigma_{s2}\right)}{\sigma_{s2}+2\sigma_{bf}+\phi_{s2}\left(\sigma_{bf}-\sigma_{s2}\right)}$,
	where $\sigma =\frac{\sigma_{s}}{\sigma_{f}}$	where $\frac{\sigma_{bf}}{\sigma_{f}}=\frac{\sigma_{s1}+2\sigma_{f}-2\phi_{s1}\left(\sigma_{f}-\sigma_{s1}\right)}{\sigma_{s1}+2\sigma_{f}+\phi_{s1}\left(\sigma_{f}-\sigma_{s1}\right)}$

**Table 3:** Estimations of error for f(η) for λ=1 (suction) by setting $S=-1.0,\;M=0.0,\;\phi_{s1}=0.0,\;$$\phi_{s2}=0.0,\;Ec=0.0$.

η	${\bar{f}}_{\mathrm{SLCM}}\left(\eta \right)$	$f_{\mathrm{exact}}\left(\eta \right)$	Error (${\bar{f}}_{\mathrm{SLCM}}\left(\eta \right)-f_{\mathrm{exact}}\left(\eta \right)$)
1	1.00000000000000	1.00000000000000	0.00
2	0.36789193242404	0.36789186311950	−6.93 $\;\times \;10^{-8}$
3	0.13536146546879	0.13536147292371	7.45$\;\times \;10^{-9}$
4	0.04982204775547	0.04982208251283	3.48$\;\times \;10^{-8}$
5	0.01835606631535	0.01835608972335	2.34$\;\times \;10^{-8}$
6	0.00678193587865	0.00678196130731	2.54$\;\times \;10^{-8}$
7	0.00252529235423	0.00252532134904	2.90$\;\times \;10^{-8}$
8	0.00096040804169	0.00096043867240	3.06$\;\times \;10^{-8}$
9	0.00038563036786	0.00038566142781	3.11$\;\times \;10^{-8}$
10	0.00017498845005	0.00017502073958	3.23$\;\times \;10^{-8}$

**Table 4:** Estimations of error for f(η) for λ=−1 (injection) by setting $S=-1.0,\;M=0.0,\;\phi_{s1}=0.0,\;$$\phi_{s2}=0.0,\;Ec=0.0$.

η	${\bar{f}}_{\mathrm{SLCM}}\left(\eta \right)$	$f_{\mathrm{exact}}\left(\eta \right)$	Error (${\bar{f}}_{\mathrm{SLCM}}\left(\eta \right)-f_{\mathrm{exact}}\left(\eta \right)$)
1	−0.94885743676949	−0.94885743745418	−6.85 $\;\times \;10^{-10}$
2	−1.63455312624474	−1.63455316937937	−4.31 $\;\times \;10^{-8}$
3	−1.78885923364456	−1.78885936169021	−1.28 $\;\times \;10^{-7}$
4	−1.59588126180011	−1.59588141160492	−1.50 $\;\times \;10^{-7}$
5	−1.26851280672842	−1.26851297968553	−1.73 $\;\times \;10^{-7}$
6	−0.91665717943458	−0.91665739473288	−2.15 $\;\times \;10^{-7}$
7	−0.58133707777713	−0.58133732132217	−2.44 $\;\times \;10^{-7}$
8	−0.27287457067674	−0.27287483123303	5.46$\;\times \;10^{-7}$
9	0.00922781670940	0.00922754203252	−2.75 $\;\times \;10^{-7}$
10	0.26829757023230	0.26829728193448	−2.88 $\;\times \;10^{-7}$

**Table 5:** Estimations of error for $f^{\prime }\left(\eta \right)$ for λ=1 (suction) by setting $S=-1.0,\;M=0.0,\;\phi_{s1}=0.0,\;$$\phi_{s2}=0.0,\;Ec=0.0$.

η	${\bar{f^{\prime }}}_{\mathrm{SLCM}}\left(\eta \right)$	$f_{\mathrm{numerical}}^{\prime }\left(\eta \right)$	Error (${\bar{f^{\prime }}}_{\mathrm{SLCM}}\left(\eta \right)-f_{\mathrm{numerical}}^{\prime }\left(\eta \right)$)
1	−1.00000000000000	−1.00000000000000	0.00
2	−0.36786358638700	−0.36786349969459	8.67$\;\times \;10^{-8}$
3	−0.13532428572200	−0.13532412458892	1.61$\;\times \;10^{-7}$
4	−0.04978027160060	−0.04978024837351	2.32$\;\times \;10^{-8}$
5	−0.01831132674283	−0.01831133136718	−4.64 $\;\times \;10^{-9}$
6	−0.00673498525720	−0.00673498845107	−3.19 $\;\times \;10^{-9}$
7	−0.00247653098871	−0.00247653141960	−4.31 $\;\times \;10^{-10}$
8	−0.00091009345899	−0.00091009280523	6.54$\;\times \;10^{-10}$
9	−0.00033394883843	−0.00033394936560	−5.27 $\;\times \;10^{-10}$
10	−0.00012208767144	−0.000122088679195	−1.01 $\;\times \;10^{-9}$

**Table 6:** Estimations of error for $f^{\prime }\left(\eta \right)$ for λ=−1 (injection) by setting $S=-1.0,\;M=0.0,\;\phi_{s1}=0.0,\;$$\phi_{s2}=0.0,\;Ec=0.0$.

η	${\bar{f^{\prime }}}_{\mathrm{SLCM}}\left(\eta \right)$	$f_{\mathrm{numerical}}^{\prime }\left(\eta \right)$	Error (${\bar{f^{\prime }}}_{\mathrm{SLCM}}\left(\eta \right)-f_{\mathrm{numerical}}^{\prime }\left(\eta \right)$)
1	−0.881658216000000	−0.881658160131000	5.59$\;\times \;10^{-8}$
2	−0.413472954408000	−0.413473033780900	−7.94 $\;\times \;10^{-8}$
3	0.062800133457000	0.062799921742630	−2.12 $\;\times \;10^{-7}$
4	0.286662267700000	0.286662236841000	−3.09 $\;\times \;10^{-8}$
5	0.350604233706000	0.350604192538500	−4.12 $\;\times \;10^{-8}$
6	0.346923293430430	0.346923259594730	−3.38 $\;\times \;10^{-8}$
7	0.322305177358000	0.322305154823200	−2.25 $\;\times \;10^{-8}$
8	0.294820060332000	0.294820047967200	−1.24 $\;\times \;10^{-8}$
9	0.269976487665000	0.269976472735700	−1.49 $\;\times \;10^{-8}$
10	0.248753377628000	0.248753360146500	−1.75 $\;\times \;10^{-8}$

**Table 7:** Estimations for $f^{\prime \prime }\left(1\right)$ for λ with Zaimi et al. [6] by setting $S=-1.0,\;M=0.0,\;\phi_{s1}=0.0,\;$$\phi_{s2}=0.0,\;Ec=0.0$.

λ	Zaimi et al. [6]	Present Result
1.0	1.00089	0.9999997414
1.5	1.91766	1.9176290901
2.0	2.56321	2.5632048156
3.0	3.70205	3.7020564530
4.0	4.77219	4.7721934962
5.0	5.81516	5.8151611668
6.0	6.84433	6.8443346713

**Table 8:** Estimation of $f^{\prime \prime }\left(1\right)$ and $\theta^{\prime }\left(1\right)$ for different physical parameters.

Parameters	$\phi_{s1}$	$\phi_{s2}$	$f^{\prime }\left(1\right)$		$\theta^{\prime }\left(1\right)$	
			Suction	Injection	Suction	Injection
	0	0	−2.45157438	−1.00000409	60.68585714	6.26887639
M = 0,	0	0.05	−2.53312395	−1.01711617	54.63809435	5.94646811
Ec = 0	0.05	0	−2.55626466	−1.02191482	53.44062750	5.88037379
	0.05	0.05	−2.57050006	−1.02485456	48.33622616	5.59179937
	0	0	−2.64176799	−1.25586249	42.10990669	1.77458537
M = 0.5,	0	0.05	−2.72109157	−1.27252911	35.03251455	1.11696813
Ec = 0.5	0.05	0	−2.74272031	−1.27602727	34.11850212	1.10939540
	0.05	0.05	−2.75926448	−1.28243658	28.35877227	0.52519882

## Data Availability

All data are available within the text.

## Abbreviations

The following abbreviations are used in this manuscript:

PDEs	Partial Differential Equations
ODEs	Ordinary Differential Equations
SLCM	Shifted Legendre Collocation Method
a(t)	Unsteady radius of the cylinder
$a_{0}$	Positive constant
$B_{0}$	Magnetic field intensity
$C_{p}$	Specific heat [J/kg·K]
$C_{fx}$	Skin friction coefficient
Ec	Eckert number
*f*	Dimensionless stream function
*k*	Thermal conductivity
$L_{n}$	Legendre polynomial
*M*	Magnetic parameter
*n*	Degree of the polynomial
$Nu_{x}$	Local Nusselt number
*P*	Pressure [Pa]
Pr	Prandtl number of base fluid
$q_{w}$	Wall heat flux [W/m${}^{2}]$
$Re_{x}$	Local Reynolds number
*r*	Coordinates measured in the radial direction
*t*	Time
*T*	Local fluid temperature [K]
$T_{w}$	Temperature at the surface of the sheet [K]
$T_{\infty }$	Free stream temperature [K]
*u*	*r*-component of velocity [m/s]
*U*	Suction velocity [m/s]
*w*	*z*-component of velocity [m/s]
*z*	Coordinates measured in the axial direction
**Greek Letters**	
α	Thermal diffusivity [m${}^{2}$/s]
γ	Constant
ϕ	Volume fraction of nanofluid
η	Similarity variable
λ	Mass flux parameter
μ	Absolute viscosity [Ns/m${}^{2}]$
ν	Kinematic viscosity [m${}^{2}$/s]
σ	Electric conductivity [S/m]
ρ	Density [kg/m${}^{3}]$
θ	Dimensionless temperature
ψ	Stream function
$\tau_{w}$	Viscous stress at the surface [Nm${}^{-2}$]
**Subscripts**	
hnf	Hybrid nanofluid
bf,f	Base fluid
s1,s2	Solid nanoparticles
*∞*	Boundary layer edge
**Superscripts**	
${}^{\prime }$	Differentiation with respect to η

## References

[1] Dinarvand S., Rostami M.N., Dinarvand R., Pop I. (2019). Improvement of drug delivery micro-circulatory system with a novel pattern of CuO-Cu/blood hybrid nanofluid flow towards a porous stretching sheet. Int. J. Numer. Methods Heat Fluid Flow.

[2] Vuong T.K.O., Le T.T., Do H.D., Nguyen X.T., Nguyen X.C., Vu T.T., Le T.L. (2020). PMAO-assisted thermal decomposition synthesis of high-stability ferrofluid based on magnetite nanoparticles for hyperthermia and MRI applications. Mater. Chem. Phys..

[3] Qi C., Tang J., Fan F., Yan Y. (2020). Effects of magnetic field on thermo-hydraulic behaviors of magnetic nanofluids in CPU cooling system. Appl. Therm. Eng..

[4] Fan F., Qi C., Tang J., Liu Q., Wang X., Yan Y. (2020). A novel thermal efficiency analysis on the thermo-hydraulic performance of nanofluids in an improved heat exchange system under adjustable magnetic field. Appl. Therm. Eng..

[5] Mohamed M.K.A., Ismail N.A., Hashim N., Shah N.M., Salleh M.Z. (2019). MHD slip flow and heat transfer on stagnation point of a magnetite Fe_3_O_4_ ferrofluid towards a stretching sheet with Newtonian heating. CFD Lett..

[6] Zaimi K., Ishak A., Pop I. (2014). Unsteady flow due to a contracting cylinder in a nanofluid using Buongiorno’s model. Int. J. Heat Mass Transf..

[7] Elnajjar E.J., Al-Mdallal Q.M., Allan F.M. (2016). Unsteady flow and heat transfer characteristics of fluid flow over a shrinking permeable infinite long cylinder. J. Heat Transfer.

[8] Al Sakkaf L.Y., Al-Mdallal Q.M., Al Khawaja U. (2018). A numerical algorithm for solving higher-order nonlinear BVPs with an application on fluid flow over a shrinking permeable infinite long cylinder. Complexity.

[9] Al-Mdallal Q., Aman S., Al Fahel S., Dadoa S., Kreishan T. (2019). Numerical study of unsteady flow of a fluid over shrinking long cylinder in a porous medium undermagnetic force. J. Nanofluids.

[10] Saranya S., Al-Mdallal Q.M. (2020). Non-Newtonian ferrofluid flow over an unsteady contracting cylinder under the influence of aligned magnetic field. Case Stud. Therm. Eng..

[11] Hosseinzadeh K., Asadi A., Mogharrebi A.R., Azari M.E., Ganji D.D. (2021). Investigation of mixture fluid suspended by hybrid nanoparticles over vertical cylinder by considering shape factor effect. J. Therm. Anal. Calorim..

[12] Abbas N., Nadeem S., Saleem A., Malik M.Y., Issakhov A., Alharbi F.M. (2021). Models base study of inclined MHD of hybrid nanofluid flow over nonlinear stretching cylinder. Chin. J. Phys..

[13] Sundar L.S., Sharma K.V., Singh M.K., Sousa A.C.M. (2017). Hybrid nanofluids preparation, thermal properties, heat transfer and friction factor-a review. Renew. Sustain. Energy Rev..

[14] Sajid M.U., Ali H.M. (2018). Thermal conductivity of hybrid nanofluids: A critical review. Int. J. Heat Mass Transf..

[15] Devi S.U., Devi S.A. (2017). Heat transfer enhancement of Cu-Al_2_O_3_/Water hybrid nanofluid flow over a stretching sheet. J. Nigerian Math. Soc..

[16] Usman M., Hamid M., Zubair T., Haq R.U., Wang W. (2018). Cu-Al_2_O_3_/Water hybrid nanofluid through a permeable surface in the presence of nonlinear radiation and variable thermal conductivity via LSM. Int. J. Heat Mass Transf..

[17] Maskeen M.M., Zeeshan A., Mehmood O.U., Hassan M. (2019). Heat transfer enhancement in hydromagnetic alumina-copper/water hybrid nanofluid flow over a stretching cylinder. J. Therm. Anal. Calorim..

[18] Nadeem S., Abbas N. (2019). On both MHD and slip effect in micropolar hybrid nanofluid past a circular cylinder under stagnation point region. Can. J. Phys..

[19] Khashi’ie N.S., Arifin N.M., Hafidzuddin E.H., Wahi N. (2019). Thermally stratified flow of *C**u*-*A**l*_2_*O*_3_/water hybrid nanofluid past a permeable stretching/shrinking circular cylinder. J. Adv. Res. Fluid Mech. Therm. Sci..

[20] Aminian E., Moghadasi H., Saffari H. (2020). Magnetic field effects on forced convection flow of a hybrid nanofluid in a cylinder filled with porous media: A numerical study. J. Therm. Anal. Calorim..

[21] Kumar K.A., Sandeep N., Sugunamma V., Animasaun I.L. (2020). Effect of irregular heat source/sink on the radiative thin film flow of MHD hybrid ferrofluid. J. Therm. Anal. Calorim..

[22] Giwa S.O., Sharifpur M., Meyer J.P. (2020). Effects of uniform magnetic induction on heat transfer performance of aqueous hybrid ferrofluid in a rectangular cavity. Appl. Therm. Eng..

[23] Suleman M., Ramzan M., Ahmad S., Lu D., Muhammad T., Chung J.D. (2019). A numerical simulation of Silver-Water nanofluid flow with impacts of Newtonian heating and homogeneous-heterogeneous reactions past a nonlinear stretched cylinder. Symmetry.

[24] Li Z., Shafee A., Kandasamy R., Ramzan M., Al-Mdallal Q.M. (2019). Nanoparticle transportation through a permeable duct with Joule heating influence. Microsyst. Technol..

[25] Kandwal S., Mishra A., Kumar M. (2019). Numerical investigation of nanofluid heat transfer in an inclined stretching cylinder under the influence of suction/injection and viscous dissipation. Nanosci. Technol. Int. J..

[26] Mishra A., Kumar M. (2019). Ohmic-Viscous dissipation and heat generation/absorption effects on MHD nanofluid flow over a stretching cylinder with suction/injection. Adv. Intell. Syst. Comput..

[27] Khashi’ie N.S., Arifin N.M., Nazar R., Hafidzuddin E.H., Wahi N., Pop I. (2020). Magnetohydrodynamics (MHD) axisymmetric flow and heat transfer of a hybrid nanofluid past a radially permeable stretching/shrinking sheet with joule heating. Chin. J. Phys..

[28] Aly E.H., Pop I. (2020). MHD flow and heat transfer near stagnation point over a stretching/shrinking surface with partial slip and viscous dissipation: Hybrid nanofluid versus nanofluid. Powder Technol..

[29] Chamkha A.J., Dogonchi A.S., Ganji D.D. (2019). Magneto-hydrodynamic flow and heat transfer of a hybrid nanofluid in a rotating system among two surfaces in the presence of thermal radiation and Joule heating. AIP Adv..

